# Relationship between Contact Pressure and Motion Artifacts in ECG Measurement with Electrostatic Flocked Electrodes Fabricated on Textile

**DOI:** 10.1038/s41598-019-42027-x

**Published:** 2019-04-11

**Authors:** Toshihiro Takeshita, Manabu Yoshida, Yusuke Takei, Atsushi Ouchi, Akinari Hinoki, Hiroo Uchida, Takeshi Kobayashi

**Affiliations:** 1 National Institute of Advanced Industrial Science and Technology (AIST), Research Center for Ubiquitous MEMS and Micro Engineering, Tsukuba, 305-8564 Japan; 2 National Institute of Advanced Industrial Science and Technology (AIST), Flexible Electronics Research Center, Tsukuba, 305-8564 Japan; 3 Nagoya university, Graduate School of Medicine, Nagoya, 466−8550 Japan

## Abstract

To develop a wearable multi-lead electrocardiogram (ECG) measuring system, we fabricated the electrodes and wires by using electrostatic flocking technology on a textile. By using this technology, it was possible to fabricate many electrodes and wires, simultaneously. Also the flocked electrodes and wires had stretchability and washing resistance properties. To use dry electrodes, it is important to reduce the influence of motion artifacts (MAs). The results of the experiment with the skin phantom revealed that the contact pressure between the skin and the electrode is an important factor in MA reduction. Then, we conducted experiments with a human body to determine the relationship between the contact pressure and the MAs. Under the pressures of 200 Pa and 500 Pa, MAs were observed. Meanwhile, under the pressures of 1000 Pa, 2000Pa and 4000 Pa, the ECG signals under rest and deep breathing conditions were able to be measured without MAs. Considering the comfortability, the contact pressure from 1000 Pa to 2000 is preferable. Finally, we fabricated the wearable ECG measuring system and succeeded in measuring 18-lead ECG signals. The measured ECG waveform is in good agreement with the ECG waveform measured by a commercial system.

## Introduction

Wearable vital sign measurement systems are attracting worldwide attention because they have significant benefits as medical and healthcare applications. For example, various developed wearable sensors, such as electrocardiogram (ECG)^1,2^, electromyogram (EMG)^3,4^, pulse wave^5,6^, blood flow^7,8^, and temperature^9–11^ sensors have been reported. In particular, considering that the leading cause of death in the world is heart disease, the demand for wearable ECG measurement equipment is very high^12^. However, existing wearable ECG measurement equipment has only one or two leads^13–16^. Because the amount of information is not enough to accurately diagnose heart disease, these ECG devices have been developed for monitoring or sports applications and not for medical applications. At a minimum, the measurement of 12-lead ECG signals, which is generally performed during a common medical checkup is required for medical applications. In this study, our objective was to measure 12-lead ECG signals by using a wearable ECG measuring system to realize long time and stress-free measurement in hospital for medical applications.

When measuring a 12-lead ECG signal, it is necessary to attach at least ten electrodes (four electrodes on the limb, and six electrodes around the chest). In the case of the proposed wearable textile ECG measuring system, the electrodes were fabricated separately and were attached to the textile. Therefore, when the number of electrodes increased, the fabrication became complicated. Thus, we developed a wearable multi-lead ECG measuring system, which was fabricated by using electrostatic flocking technology. With the electrostatic flocking technology, fibers are sprayed onto textiles where the adhesive paste is printed, and the fibers form on the textile in a standing state against the textile, vertically^17^. We fabricated the electrodes and wires on the textile by using technologies that enable the batch fabrication of many electrodes and wires. Rai *et al*. had reported the development of a dry electrode and a patch electrode by the electrostatic flocking technology^18,19^. Moreover, they succeed in measuring ECG signals with the flocked electrode.

However, motion artifacts (MAs) are a big problem, not only for flocked electrodes but also for many types of dry electrodes, because contact between the skin and the dry electrode is unstable. Therefore, in a dry electrode, the surface of the electrode and the design of the electrode’s mechanical properties are important in MAs reduction, which has been previously achieved by providing cushioning^20–22^ to a dry electrode, or by sticking the electrode into the skin with a small needle^23–25^. However, in the case of a wearable measurement system, not only the surface and mechanical properties, but also contact pressure is significant. Even if good mechanical contact is obtained, MAs may still occur because of the displacement between the electrode and the skin, when the contact pressure of the wearable system is insufficient.

In this paper, the relationship between the contact pressure and the MAs, when using the flocked electrode, was investigated by implementing two experimental setups, which used a skin phantom and the human body, respectively. In our case, because the wearable ECG measuring system is intended for use in hospital, the patient is relaxed and doesn’t move much. Therefore, we can focus on reducing the MAs caused by breathing. From these experimental results, we determined the contact pressure required between the skin and the flocked electrodes to measure the ECG signals without MAs. Finally, we fabricated the wearable multi-lead ECG measuring system and measured the 12-ECG signals.

## Results

### Electrostatic flocking technology

**Figure 1:**
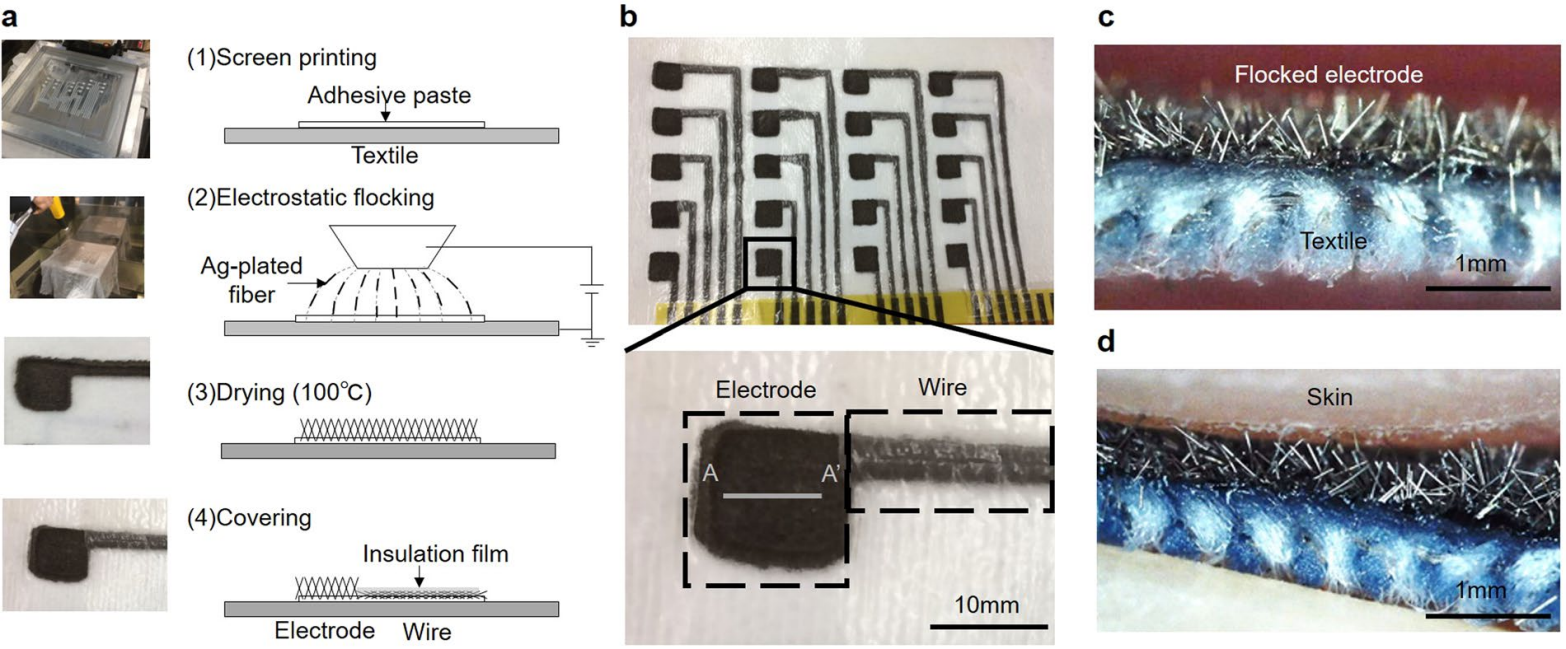
Flocked electrodes and wires fabricated by electrostatic flocking technology. (**a**) A fabrication process of electrodes and wires using electrostatic flocking technology. Ag-plated fiver was flocked on textile with adhesive paste. (**b**) Photo of electrodes and wires fabricated on textile. (**c**) Cross section view (A-A′ line) of electrode (**d**) when skin touched the electrode.

Figure 1(a) shows the fabrication of electrodes and wires by using electrostatic flocking technology. After printing the adhesive paste on the textile, Ag-plated fiber was formed by electrostatic flocking technology, which charges the fiber (polyester) and sprays it along the electric field that aligns the charge fibers vertically. The length of the fiber was 500 μm, and its diameter was 18 μm. The details of the fabrication process are described in later sections. Subsequently, the adhesive paste was dried. Finally, the wiring part was covered with insulating film. Figure 1(b) shows the flocked electrodes and wires. By using flocking technology, many electrodes and wires can be fabricated at a low cost. Additionally, Fig. 1(c,d) shows a cross-sectional view of the flocked electrode and the flocked electrode in contact with the skin. The edge of the Ag-plated fiber caught the surface of the skin and produced a high frictional force. Even if the position of the skin was moved because of body movement, such as breathing, for example, the electrode could follow the movement when contact pressure was sufficient. Consequently, it was possible to reduce the influence of MAs.

### Evaluation of Electrostatic flocked wire

**Figure 2:**
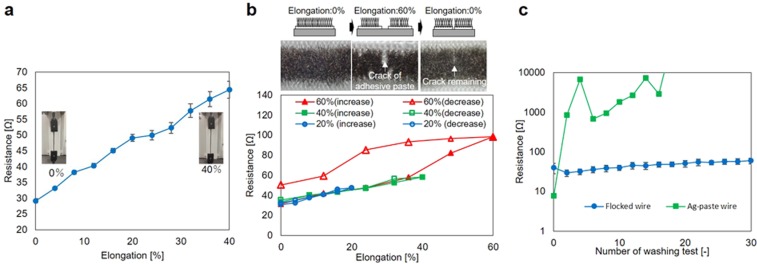
Evaluation of stretchability and wash resistance flocked wires. (**a**) Mean value and standard error (five samples) of resistance when the flocked wire sample was tensile to 40%. (**b**) Hysteresis property and recovery property of flocked wire. (**c**) Washing resistance of the flocked wire and Ag-paste wire. The washing test was conducted according to a standard washing test (JIS L 1930 C4N 140). The graph shows the mean values and standard errors (two samples) of resistance.

First, measurement experiments were conducted to evaluate the stretchability and washing resistance of the flocked wire. Figure 2(a) presents the experimental results obtained by a tensile test with the flocked wire. The wire length of the test sample was 60 mm, and its width was 3 mm. The width was determined by the fineness of the adhesive paste printing process. The resistance value of the flocked wire before the tensile test was 29.1 Ω. After applying 40% elongation, the resistance value increased to 64.3 Ω. The change of resistance was 35.3 Ω. However, considering that the contact resistance between the skin-electrode amounts to a few hundred kΩ, the resistance change was small enough to measure the ECG signal. Figure 2(b) shows hysteresis property and recovery property of the flocked wire. The three test samples were prepared for 20%, 40%, and 60% elongation test, respectively. When the elongation was smaller than 40%, the resistance of the flocked wire did not have hysteresis property, and the resistance value was recovered when the elongation was released. The resistance value of before and after 20% elongation were 30.6 Ω and 32.4 Ω. Also, the resistance value of before and after 40% elongation were 33.3 Ω and 35.4 Ω. However, the hysteresis property appeared, and the resistance value was not recovered when the elongation was 60%. The resistance value of before and after 60% elongation was 31.5 Ω and 50.4 Ω. However, because the elongation of the upper body, which is caused by motion and wearing, is 40% or less, it is thought that the 40% elongation tolerance is large enough for actual use. The results are discussed in the discussion section. Figure 2(c) shows the resistance change in 30 times standard washing test. The flocked wire resistance was larger than the resistance of the Ag-paste wire, initially. However, the resistance of the Ag-paste fiber increased with the number of washing tests. Otherwise, the change in flocked wire resistance was small. The resistance change was small enough to measure ECG. As a result of this experiment, the flocked wire has wash resistance.

### Evaluation of Electrostatic flocked electrode

Next, characteristics of flocked electrode against MAs was evaluate by experiments. The influence of MAs (V_ma_) is expressed as follows^26^.

1 $${{\rm{V}}}_{{\rm{ma}}}=2[{{\rm{\Delta }}{\rm{V}}}_{{\rm{dc}}-{\rm{offset}}}+{{\rm{\Delta }}{\rm{Z}}}_{{\rm{skin}}-{\rm{electrode}}}(\frac{(\frac{{{\rm{V}}}_{{\rm{ECG}}}}{2})+{{\rm{V}}}_{{\rm{dc}}-{\rm{offset}}}}{{{\rm{Z}}}_{{\rm{in}}}}+{{\rm{I}}}_{{\rm{bias}}})]$$

In this equation, V_dc − offset_, Z_skin − electrode_, V_ECG_, and I_baias_ represent the offset voltage at the skin-electrode interface, impedance between the skin and the electrode, ECG signal voltage, and input impedance of the amplifier, respectively. ΔV_dc − offset_ and ΔZ_skin − electrode_ are major causes of MAs. In this study, we focused on ΔZ_skin − electrode_ because the flocked electrode was dry electrode. Therefore, we experimentally determined how much contact pressure was necessary to reduce V_ma_.

#### Results of experiment with skin phantom

**Figure 3:**
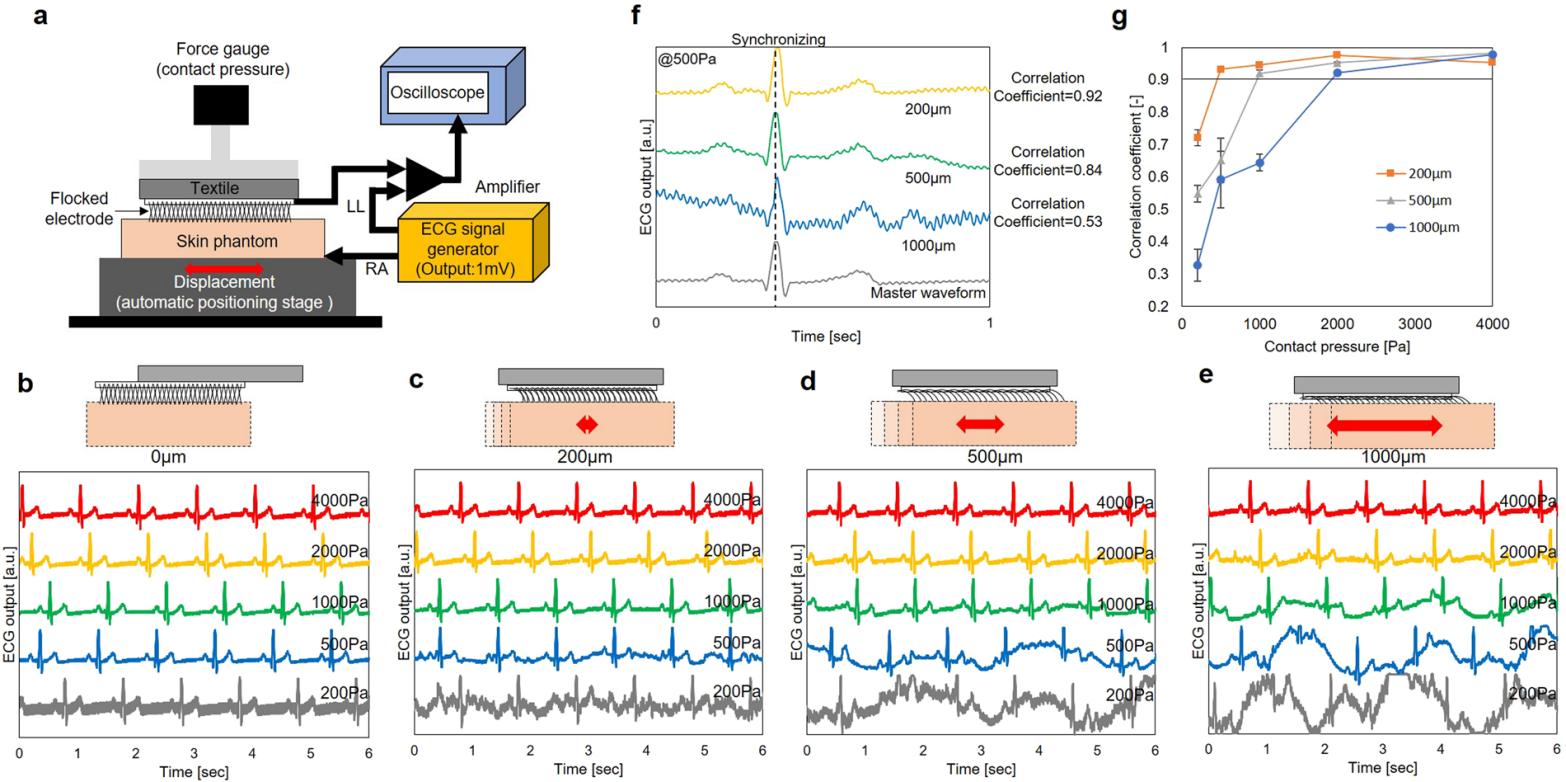
Evaluation of flocked electrodes using skin phantom. (**a**) Image of the experimental setup. ECG signals which were inputted by ECG signal generator and measured by using skin phantom when the contact pressure between skin phantom and flocked electrode was 200 Pa, 500 Pa, 1000 Pa, 2000 Pa, and 4000 Pa. The displacement of the skin phantom was (**b**) 0 µm, (**c**) 200 µm, (**d**) 500 µm, and (**e**) 1000 µm. **(f**) Example of the calculation result of correlation coefficients with contact pressure of 500 Pa and with displacement of 200 µm, 500 µm, and 1000 µm. (**g**) Mean value and standard error (five samples) of the correlation coefficient of ECG signals against master waveform when the contact pressure was 200 Pa, 500 Pa, 1000 Pa 2000 Pa, and 4000 Pa.

First, the skin phantom used in the experiment had mechanical characteristics, which were identical to the characteristics of the human skin. The experimental system is shown in Fig. 3(a), and the experimental setup is described in detail in the method section. The skin phantom was connected to the ECG signal generator, and an electrode was in contact with the skin phantom. The ECG signal measured by the electrode was evaluated when the skin phantom moved or when the contact pressure was changed. The frequency of the stage was 14 round trips/minute (equivalent to the human breathing frequency). The contact pressures were 200 Pa, 500 Pa, 1000 Pa, 2000 Pa, and 4000 Pa. The size of the flocked electrode was 10 mm × 10 mm. Figure 3(b–e) shows the ECG signal under each pressure condition, when the displacement of the stage was 0 µm (not moving), 200 μm (shoter than the length of the Ag-fiber), 500 μm (identical to the length of the Ag-fiber length), and 1000 μm (longer than the length of the Ag-fiber) under each pressure condition, respectively. The correlation coefficients were calculated to estimate the minimum contact pressure to measure ECG signals without MAs. The calculation was carried out as follows:

1. Extract five waveforms for 1 second from the ECG signal, when the displacement of the skin phantom is 0 µm. Next, the average of the five waveforms. The averaged waveform is defined as the master waveform for a specific contact pressure.
2. Extract five waveforms under the specific contact pressure for 1 second, when the displacement of the skin phantom is 200 µm, 500 µm, and 1000 µm, and synchronize with the master data at the position of the R wave peak.
3. Normalize the peak value such that it becomes identical with the peak of the R wave.
4. Calculate the correlation coefficient between the master waveform and the five waveforms.
5. Calculate the mean value of the five correlation coefficients.

Figure 3(f) shows the calculation results with contact pressure of 500 Pa and the displacements of 200 µm, 500 µm, and 1000 µm. The correlation coefficients were 0.53, 0.84, and 0.92, respectively. In the ECG signal of 0.53 correlation coefficient, high frequency noise of the baseline was large, and it was difficult to identify P-wave and T-wave, particularly. In the ECG signal of 0.83 correlation coefficient, P, Q, R, S and T-wave can be identified. However, the baseline had fluctuation. Finally, the ECG signal of 0.91 correlation coefficient had no fluctuation of baseline, and had good agreement with master waveform. From these results, we defined the correlation coefficient of over 0.9 as ECG signal without MAs. Figure 3(g) shows the mean value and standard error of the correlation coefficients, which depended on the contact pressure and displacement. When the contact pressure was high, or the displacement was small, the correlation coefficient became high. When the displacements were 200 μm, 500 µm, and 1000 µm, the correlation coefficients reached over 0.9 at 500 Pa, 1000 Pa, 2000 Pa, and 4000 Pa, respectively. The length of the chest difference as a result of breathing was less than 5%, even under deep breathing conditions. Because the size of the electrode was 10 mm × 10 mm, the displacement between the skin and the flocked electrode caused by breathing was estimated at the maximum value of 500 μm. Therefore, a contact pressure over 1000 Pa was desirable to obtain an ECG signal without MAs caused by breathing.

#### Results of experiment with human body

**Figure 4:**
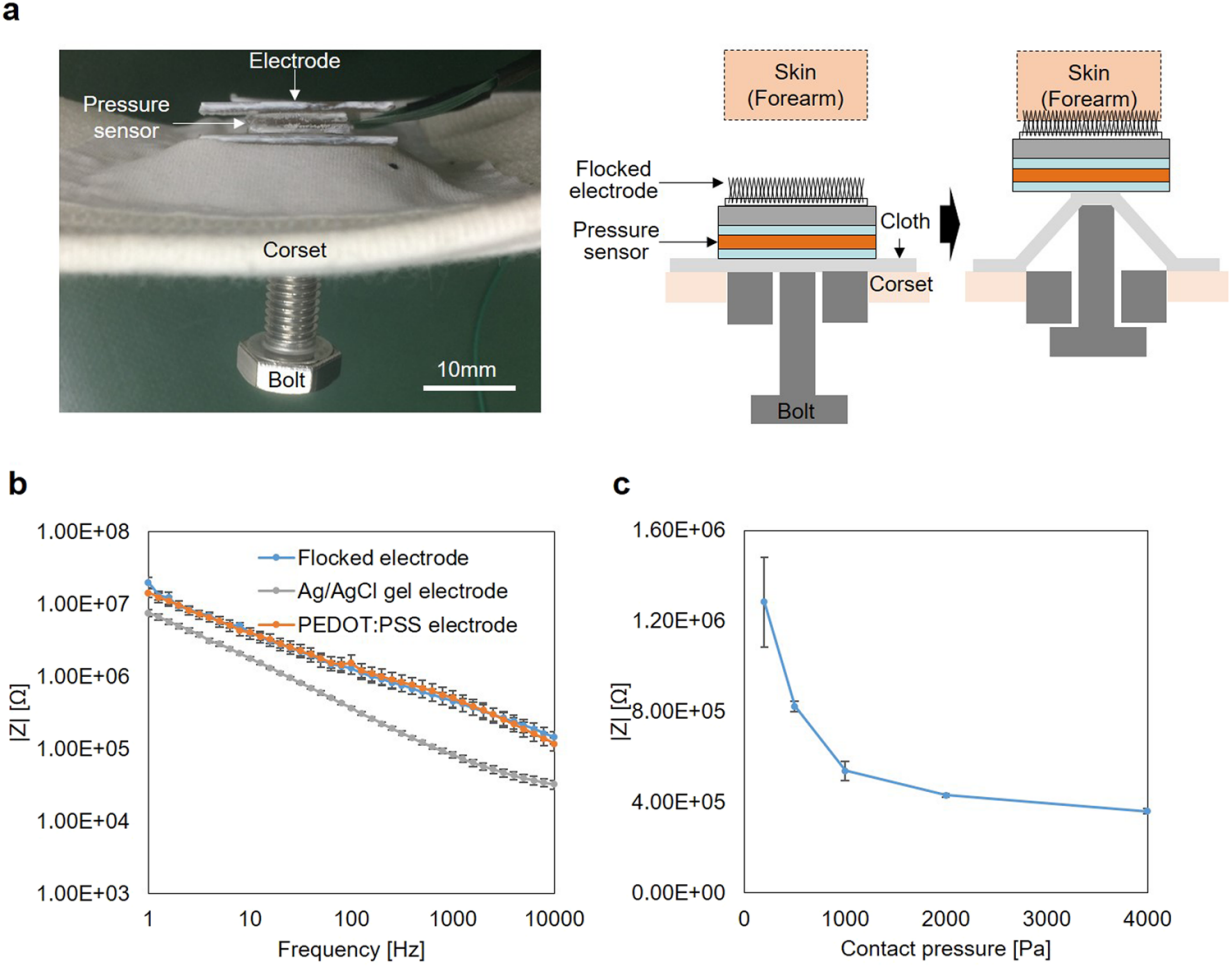
Evaluation of contact impedance of the flocked electrodes. (**a**) Experimental setup to measure impedance while changing contact pressure. (**b**) Impedance spectra of flock electrode, Ag/AgCl gel electrode, and PEDOT: PSS electrode measured in the 1 Hz to the 10 kHz frequency range in contact with skin (forearm) at 200 Pa. It shows the mean value and standard error (five Samples). (**c**) Dependence of impedance on contact pressure at the frequency of 100 Hz. It shows the mean value and standard error (five Samples).

An impedance and ECG measurement experiments with a human body were conducted to demonstrate the result of skin phantom experiment. Figure 4(a) shows the cross-sectional view of the experimental setup which can contact electrode with skin while changing contact pressure. The details of the experimental setup are described in the method section. Figure 4(b) shows the impedance of flocked electrode, poly (3,4-ethylenedioxythiophene): poly (styrenesulfonate) (PEDOT: PSS) electrode (C3fit IN-pluse, GOLDWIN INC., Japan) and Ag/AgCl electrode (Red Dot, 3M Company, U.S.A.) when the contact pressure between skin and electrode was 200 Pa. Though the impedance of the flocked electrode is bigger than the impedance of Ag/AgCl electrode, it was almost the same with the impedance of PEDOT: PSS electrode which is commercial products. Figure 4(c) shows the dependence of impedance against contact pressure. The impedance greatly depended on the contact pressure when the contact pressure was less than 1000 Pa. On the other hand, dependence became small when the contact pressure was 1000 Pa or more. From this result, if the contact pressure was kept over 1000 Pa, the impedance becomes stable.

**Figure 5:**
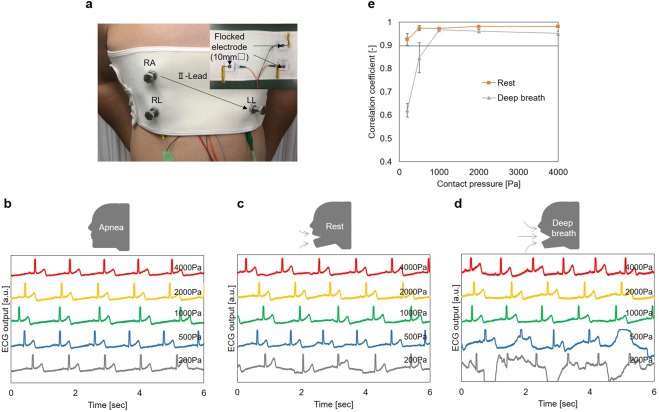
Evaluation of flocked electrodes using human body for ECG measurement. (**a**) A photo in measuring ECG signal when contact pressure was changed and cross-section view and back side of the corset ECG measurement system. ECG signals of the human body (II -Lead) when the contact pressure between the skin and flocked electrode was 200 Pa, 500 Pa, 1000 Pa, 2000Pa and 4000 Pa. The subject was (**b**) apnea, (**c**) rest and (**d**) deep breath. (**e**) Mean value and standard error of correlation coefficient value of ECG signals against master waveform when the contact pressure was 200 Pa, 500 Pa, 1000 Pa 2000 Pa, and 4000 Pa.

Figure 5(a) shows the experimental setup which can measure ECG signal while changing the contact pressure between skin and electrodes. The II-lead ECG signal was measured under apnea, rest, and deep breathing conditions and different contact pressures. The contact pressure was 200 Pa, 500 Pa, 1000 Pa, 2000 Pa, and 4000 Pa. The measurement results are presented in Fig. 5(b–d). Because the body movement was small under apnea conditions, the MAs were not observed. Moreover, obvious MAs were observed at 200 Pa under rest conditions, and at 500 Pa under deep breathing conditions. However, MAs were not observed at 1000 Pa or more. Figure 5(e) shows the mean value and standard error of the correlation coefficients calculated by the same procedure used in the skin phantom experiment. The master data were calculated by the ECG signal measured under apnea conditions. When the contact pressure was 500 Pa, the correlation coefficient was small. However, under rest and deep breathing conditions, the value of both correlation coefficients was 0.97 at 1000 Pa, which is sufficiently consistent with the master waveform. Under rest conditions, the correlation coefficient increased as the contact pressure increased. Its value was 0.98 at 4000 Pa. Conversely, under deep breathing conditions, the correlation coefficient decreased as the contact pressure increased. This was attributed to the influence of ΔV_dc − offset_. The potential of the skin depends on the skin’s depth when the electrode is pressed against the skin^26^. When the volume of the chest changes due to deep breathing, the variation of the depth of the electrode into the skin also changes under the excessive contact pressure condition. As a result, it is considered that fluctuation of the ΔV_dc − offset_ occurred as MAs. From the abovementioned results, it was concluded that pressure from 1000 Pa to 2000 Pa is desirable in ECG measurement with a flocked electrode, from the viewpoint of avoiding MAs. Additionally, concerning human discomfort caused by contact pressure, the discomfort also increased with pressure over 2000 Pa. Therefore, in terms of practical use, a contact pressure from 1000 Pa to 2000 is desirable and reasonable.

### Wearable multi-lead ECG measurement system

**Figure 6:**
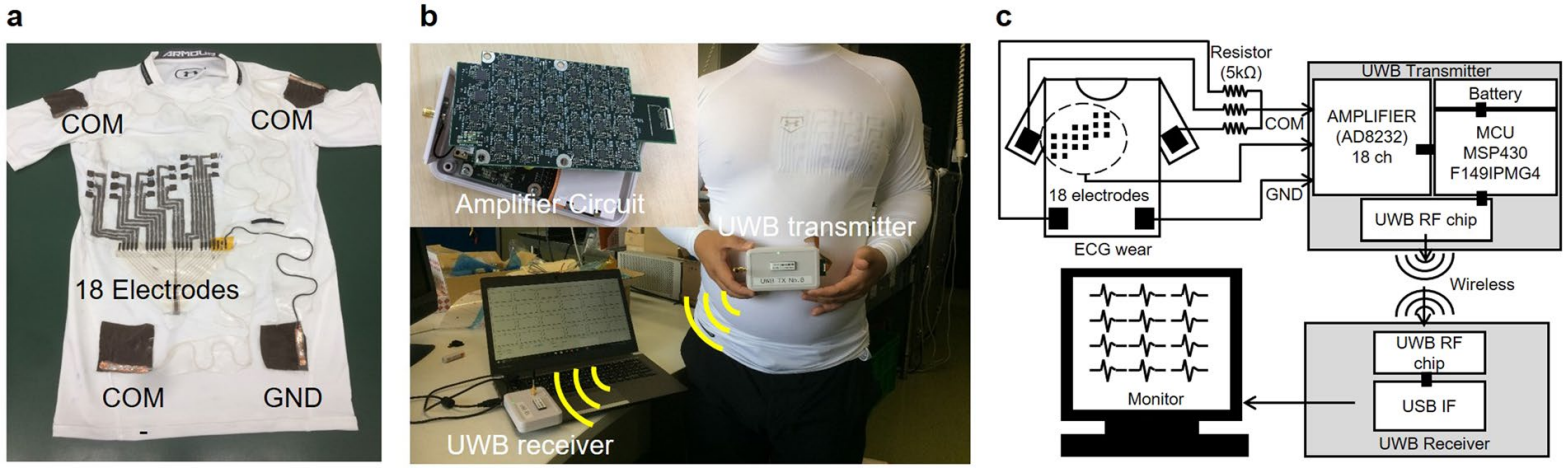
Multi-lead ECG measurement wring and system. (**a**) Photo of the multi-lead ECG measurement wear. Eighteen electrodes around the chest and four electrodes on lamb were formed by electrostatic flocking technology. (**b**) Multi-lead ECG measurement system using multi-lead ECG measurement wear and UWB transmitter. (**c**) Experimental wearable multi-lead ECG measurement system, wireless communication system, and display system.

The wearable ECG measurement system that we fabricated as a prototype is shown in Fig. 6(a). Compression wear (MCM3749, Under Armour, Inc., U.S.A.) was used as the substrate of the wearable ECG measurement system because the contact pressure of the compression wear was, approximately, from 1000 to 2000 Pa. The eighteen electrodes were placed on the left chest of the compression wear and were divided into the upper six electrodes, center six electrodes, and lower six electrodes. The positions of the center six electrodes were designed to obtain the V1–V6 signal of the Mason-Likar (ML) lead ECG signals^27^, which is equivalent to the 12-lead ECG signals. The upper six electrodes were placed at a position above the center six electrodes by one intercostal distance. Additionally, the lower six electrodes were placed at a position below the center six electrodes by one intercostal distance. The size of the chest electrode was 10 mm × 10 mm, and the width of the wiring was 3 mm. Additionally, four electrodes were formed on the limbs. The size of the limb electrode was 50 mm × 50 mm. The three electrodes placed on the left shoulder, right shoulder, and left flank, were connected through a five kΩ resistance to make a Willison center electrode (COM). The electrode placed on the right flank was a ground electrode (GND). The signal of each electrode was input to an amplifier and transmitter module through a polyimide substrate with a copper wire. In this wearable ECG measurement system, the number of electrodes was eighteen only around the chest. However, the number of electrodes can be increased easily by changing the metal mask. Figure 6(b,c) show the measurement system. A battery, amplifier circuits, and an ultra-wideband (UWB) transmission module were built in the amplifier and transmitter module. Multi-lead ECG signals were transmitted to the receiver by wireless communication and displayed on a personal computer (PC). Before measurement, the skin was cleaned with alcohol and cardio cream (Cardiocream, NIHON KIHDEN Corporation, Japan) was pasted onto the skin. The cardio cream was used to reduce time for skin and electrode to become familiar, because we couldn’t restrain the subject for a long time in terms of the ethics. Also, the medical cardio cream doesn’t have a function of adhering the electrode to the skin like a gel. Therefore, the cardio cream doesn’t irritate the skin like a gel. The measurement system is intended to use as a medical application in hospital. Therefore, using cardio cream before measuring is not unnatural or actual application. The corset was wrapped around the chest to keep the electrode in contact with the pit of the stomach. The patient sat down on a chair and relaxed.

**Figure 7:**
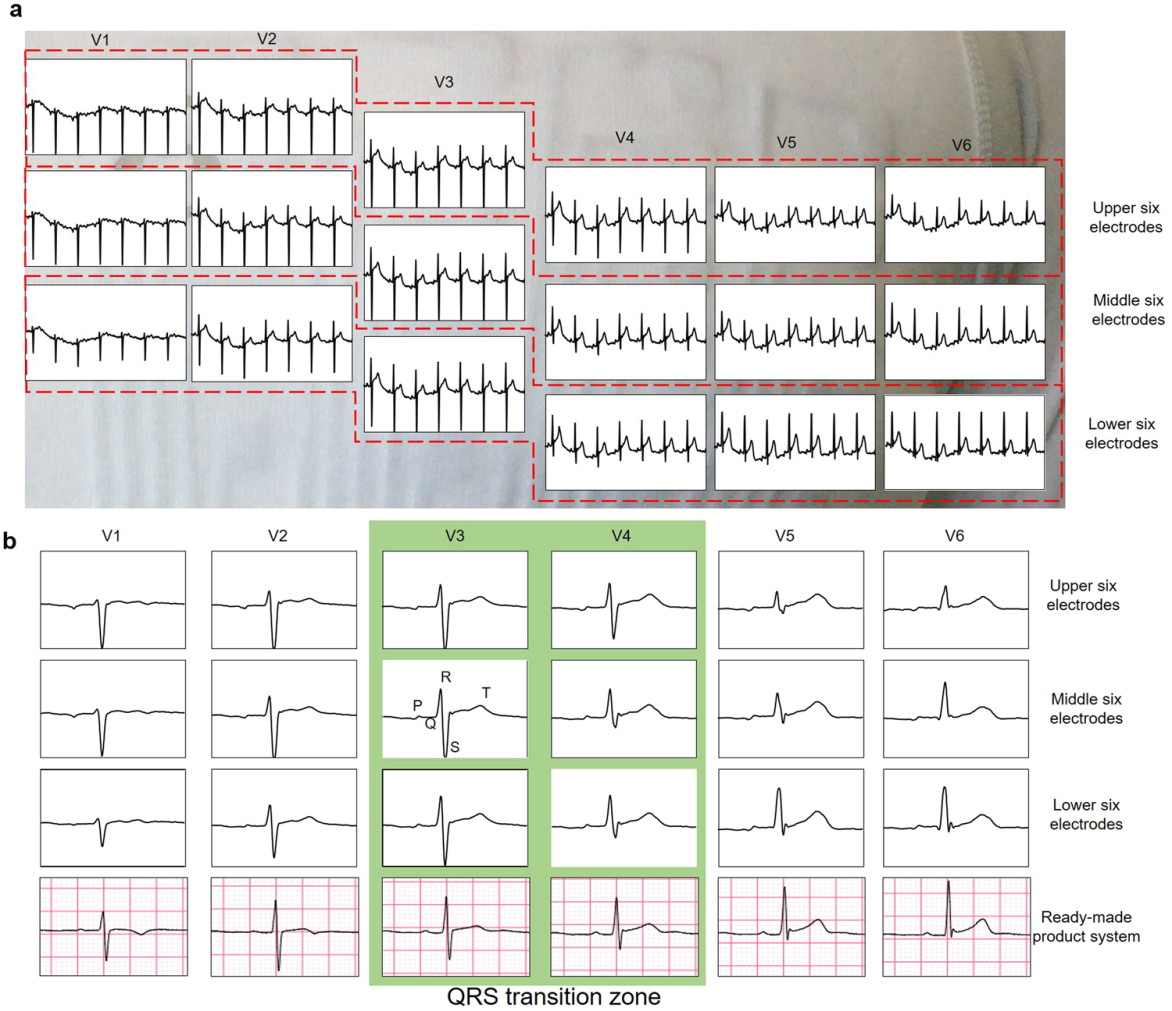
Experimental result of multi-lead ECG measurement. (**a**) 18-lead ECG signals measured by the multi-lead ECG measurement system we developed for 5 seconds. (**b**) One waveform of 18-lead ECG signals measured by the developed system and six ECG signals measured by the ready-made product system.

Figure 7(a) shows the ECG signals between the eighteen electrodes around the chest and COM for 5 seconds. Because the 18-lead ECG signals could be measured simultaneously, the multi-lead ECG measurement with the developed wearable ECG measurement was successful. However, MAs were observed in all of the signals. Because MAs in the same phase was observed in all of the 18 signals, the MAs were considered to be caused by the COM electrodes or the GND electrode attached to the limbs. Although the pressure of the eighteen electrodes was obtained sufficiently by the corset in the chest, because the four electrodes on the limbs were subjected only to clothing pressure, it was considered that the pressure was not sufficient for the four electrodes. Figure 7(b) shows the 18-lead ECG signals (from the upper six electrodes, center six electrodes, and lower six electrodes) measured by the developed system, and the six ECG signals (from the six electrodes placed on the V1–V6 positions of the 12-lead ECG) measured by an existing commercial system (SmartECG, ECG Lab Corporation, Japan). The position of the existing system’s electrode was aligned to obtain the V1–V6 signals of the common 12-lead ECG signals. First, from the signal of the eighteen electrodes, the transition zone where the height values of the R-wave and S-wave become identical, was placed between the V3 and V4 potions, which is a typical position in medical applications. Also, in comparison with the center six electrodes and upper six electrodes, the height of the V4–V6 R waves increased. In contrast, in comparison with the center six electrodes and lower six electrodes, the height of the V4–V6 R-waves decreased. Generally, the relationship between the position of the electrodes and the R-wave height that has been reported in the literature^28^ agrees with the results of the experiments conducted in this study. By comparing the ECG signals measured by the developed system with those measured by the commercial system, we found that the signal of the lower six electrodes is in good agreement with the signals measured by the commercial system. The positions of the chest electrodes (V1–V6) in the 12-lead ECG are determined by the rib structure. The ECG waveforms measured by the lower six electrodes showed the best agreement with the ECG waveforms by the commercial system because the positions of the lower six electrodes were closest to the correct electrode positions. The author (Akinari Hinoki) who is a medical doctor make the judgment of the agreement. In the wearable ECG measurement system, the positions of the electrodes depend on the body shape of the patient. However, the results demonstrate that the influence of the body shape can be reduced by fabricating many electrodes around the chest.

## Discussion

A wearable multi-lead ECG measurement system was developed by using electrostatic flocking technology. This technology uses Ag-plated fibers, which enable the fabrication of many electrodes and wiring on a textile material, simultaneously. First, the stretchability and wash resistance of the flocked wire were evaluated. When the wire extended and contracted by 40%, the resistance value of the flocked wire (length: 60 mm, width: 3 mm) changed from 29.1 Ω to 64.3 Ω. Considering that the skin contact resistance is a few hundred kΩ, the abovementioned value is sufficiently small. Moreover, after standard washing test was repeated 30 times, the resistance value of the flocked wire did not change remarkably. Thus, the results revealed that flocked wire had good stretchability and wash resistance properties. However, the flocked wire had hysteresis property as shown in Fig. 2(b). Figure 2(b) also shows the pictures of the flocked wire in 60% elongation test. The cracks of the adhesive paste appeared when the elongation reached 60%. Also, the crack remained when the elongation was returned to 0%. The conductivity of the Ag-plated fiber was realized by the many mechanical contact points between each fiber. Therefore, a large resistance change and hysteresis don’t occur as long as the contact points of the fibers are maintained. However, when the cracks occurred, the contact points of the fibers around the crack were not maintained. As a result, the large resistance changes and hysteresis occurred. In the future, the material and the thickness of adhesive paste will be optimized to prevent from cracks occurring.

Next, we evaluated the MAs of the flocked electrode. From the results of the experiment with the skin phantom, it was confirmed that the MAs depend on the amount of displacement between the skin and the electrode and the contact pressure. MAs were not observed when the contact pressure exceeded 1000 Pa, and the displacement amount was 200 µm or 500 μm. Periodic MAs is observed in Fig. 3(d,e). One reason for the periodic MAs is contact area change of flocked electrode. The skin phantom surface is not flat the same as actual skin. Contact impedance changes according to the unevenness surface of the skin phantom in the low contact pressure region, and it results in the MAs. The other reason for the periodic MAs is the length of Ag-plated fiber. When the displacement of skin phantom exceeds the fiber length (500 µm), the fiber cannot follow the displacement of skin phantom. As a result, contact impedance changes. In the moving of the reciprocating motion, the contact impedance increase. On the other hand, in stopping of reciprocating motion, the contact impedance decrease. It results in the periodic MAs. The influence of the fiber length on the contact impedance will be reported in the future.

The impedance and ECG measuring experiments with an actual human body were conducted. The impedance of the flocked electrode is depended on the contact pressure between the skin and flocked electrode. However, the dependence becomes small when the contact pressure is over 1000 Pa. This is because the fibers are sufficiently pressed against the skin at 1000 Pa. Moreover, from the result of ECG measurement experiment, it was confirmed that ECG signals without MAs were acquired, even under rest and deep breathing conditions, when the contact pressure was from 1000 Pa to 2000 Pa. The results have a good agreement with the skin phantom experiment. However, MAs were observed when the contact pressure was 4000 Pa, and the human participant breathed deeply. It was considered that MAs occurred owing to the influence of ΔV_dc − offset_ at 4000 Pa.

Finally, a wearable multi-lead ECG measurement system that can acquire 18-lead ECG signals around the chest was fabricated. The results confirm that we succeeded in simultaneously acquiring 18-lead ECG signals. Additionally, the waveform is in good agreement with the waveform obtained by the commercial ECG measurement device. This result confirms that we succeeded in acquiring the ECG waveform with sufficient medical significance. In this experiment, the flocked electrode was pressed against the skin, and a corset was used to stabilize the contact pressure between the electrode and the skin. In future work, we will optimize the wear design, the electrodes around the chest, and the electrodes on the limbs. Additionally, we will apply contact pressure from 1000 Pa to 2000 Pa for each electrode, to acquire a high-quality ECG waveform without MAs. Also, we aim to achieve higher functionality by increasing the number of electrodes. The advantage of the multi-lead ECG measurement wear is that it can be worn without discomfort even if the number of electrodes increase. We will develop ECG measurement system which is more accurately than conventional 12-lead ECG measurement system.

## Methods

The adhesive paste was mixed with the main agent (UNIBINDER NU-84, Co., Ltd., Japan) and a curing agent (CR-5L, Co., Ltd., Japan) at a ratio of 100:3. The adhesive paste was printed on the textile (MCM3749, Under Armour, Inc., USA) by using a metal mask. The thickness of the adhesive was approximately 500 μm. This adhesive paste had washing resistance and no effect on the skin. Next, electrostatic flocking was performed with Ag-plated fiber (AGposs, Mitsufuji Corporation, Japan). The length and diameter of the Ag-plating were approximately 500 μm and 17 µm, respectively. The fiber was charged to 50 kV. Subsequently, the textile flocked the Ag-plated fiber and was dried at 100 °C for 30 min. Finally, the wiring part was protected with an insulating film (Tegaderm Roll, 3 M Company, U.S.A.). In addition to its insulation properties, this film was also biocompatible.

Figure 2(a) shows the resistance change when the flocked wire was stretched by a tensile test machine (FTN1-13A, AIKOH Engineering Corporation, Japan). The number of the test sample was five. Figure 2(c) shows a resistance change of the washing test. The flocked wires (two test samples) and Ag paste wires (two test samples) were prepared for the washing test. The washing test was conducted according to the standard washing test (JIS L 1930 C4N 140) using laundry detergent (Attack powder 0.2%, Kao Corporation, Japan). After two times washing, the test sample was dried at a dry room, and the resistance of the test sample was measured. The number of washing test was 30 times, and the number of measuring resistance was 16 times including initial measurement.

Figure 3(a) shows the experimental setup used with the skin phantom (HXBNXTB858510MX, Wetlab Corporation, Japan). An ECG signal was generated by an ECG signal generator (ProSim8, Fluke Corporation, U.S.A.), which could generate ECG waveforms of several mV on the skin phantom. The right arm (RA) signal was input to the amplifier (AD8232, Analog Devices, Inc., U.S.A.) through the skin phantom and flocked the electrode. The left leg (LL) signal was input to the amplifier directly. The signal was amplified by the amplifier circuit and measured with an oscilloscope. The flocked electrode was connected to the compression test equipment (FTN1-13A, AIKOH Engineering Corporation, Japan), and the contact pressure was measured with an oscilloscope, simultaneously. The skin phantom was fixed to the automatic stage (OSMS20-35, SIGMAKOKI Co., Ltd., Japan), which was controlled by a PC, while the automatic stage moved reciprocally. When the automatic stage moved, displacement occurred between the skin phantom and the flocked electrode, and MAs appeared.

Figure 4(a) shows the experimental setup and the cross-sectional view of the experimental setup of the corset ECG measurement system. The pressure sensor (Force Sensitive Resistor 0.5, Interlink Electronics, USA) was sandwiched with the cloth, corset, and flocked electrode. There was a bolt on the back of the cloth. By using this screw, the pressure sensor and flocked electrode were pressed against the skin. The impedance was measured at forearm using potentiostat(SI 1260, Solrtron Metrology Ltd., United Kingdom) and frequency response analyzer(SI 1287, Solrtron Metrology Ltd., United Kingdom) according to the previous report^29,30^.

Then, the pressure and the ECG signal (II-lead) were measured simultaneously. Three electrodes were pressed at the same pressure, and the ECG signal was measured with an oscilloscope after being amplified. The authors declare that the material of the electrostatic flocked electrodes doesn’t have skin-irritating properties as a result of the primary skin irritation test (MTT assay), and that the all experimental protocols were approved by IRB of the ethics on the applied biomedical engineering and technology(71120030-A-20181002-001) in National Institute of Advanced Industrial Science and Technology (AIST). Moreover, the authors declare that all experiments were carried out following the relevant guidance and regulations. All volunteers provided informed signed consent to participate in the study.
